# Unexpected rip currents induced by a meteotsunami

**DOI:** 10.1038/s41598-019-38716-2

**Published:** 2019-02-14

**Authors:** Álvaro Linares, Chin H. Wu, Adam J. Bechle, Eric J. Anderson, David A. R. Kristovich

**Affiliations:** 1 Department of Civil and Environmental Engineering, University of Wisconsin-Madison, Madison, WI USA; 2 Wisconsin Sea Grant Institute, University of Wisconsin-Madison, Madison, WI USA; 3 National Oceanic and Atmospheric Administration, Great Lakes Environmental Research Laboratory, Ann Arbor, MI USA; 4 ISWS, Prairie Research Institute, University of Illinois at Urbana-Champaign, Urbana, IL USA

## Abstract

A tragic drowning event occurred along southeastern beaches of Lake Michigan on a sunny and calm July 4, 2003, hours after a fast-moving convective storm had crossed the lake. Data forensics indicates that a moderate-height (~0.3 m) meteotsunami was generated by the fast-moving storm impacting the eastern coast of the lake. Detailed Nearshore Area (DNA) modeling forensics on a high-resolution spatial *O*(*1 m*) grid reveals that the meteotsunami wave generated unexpected rip currents, changing the nearshore condition from calm to hazardous in just a few minutes and lasting for several hours after the storm. Cross-comparison of rip current incidents and meteotsunami occurrence databases suggests that meteotsunamis present severe water safety hazards and high risks, more frequently than previously recognized. Overall, meteorological tsunamis are revealed as a new generation mechanism of rip currents, thus posing an unexpected beach hazard that, to date, has been ignored.

## Introduction

**Figure 1:**
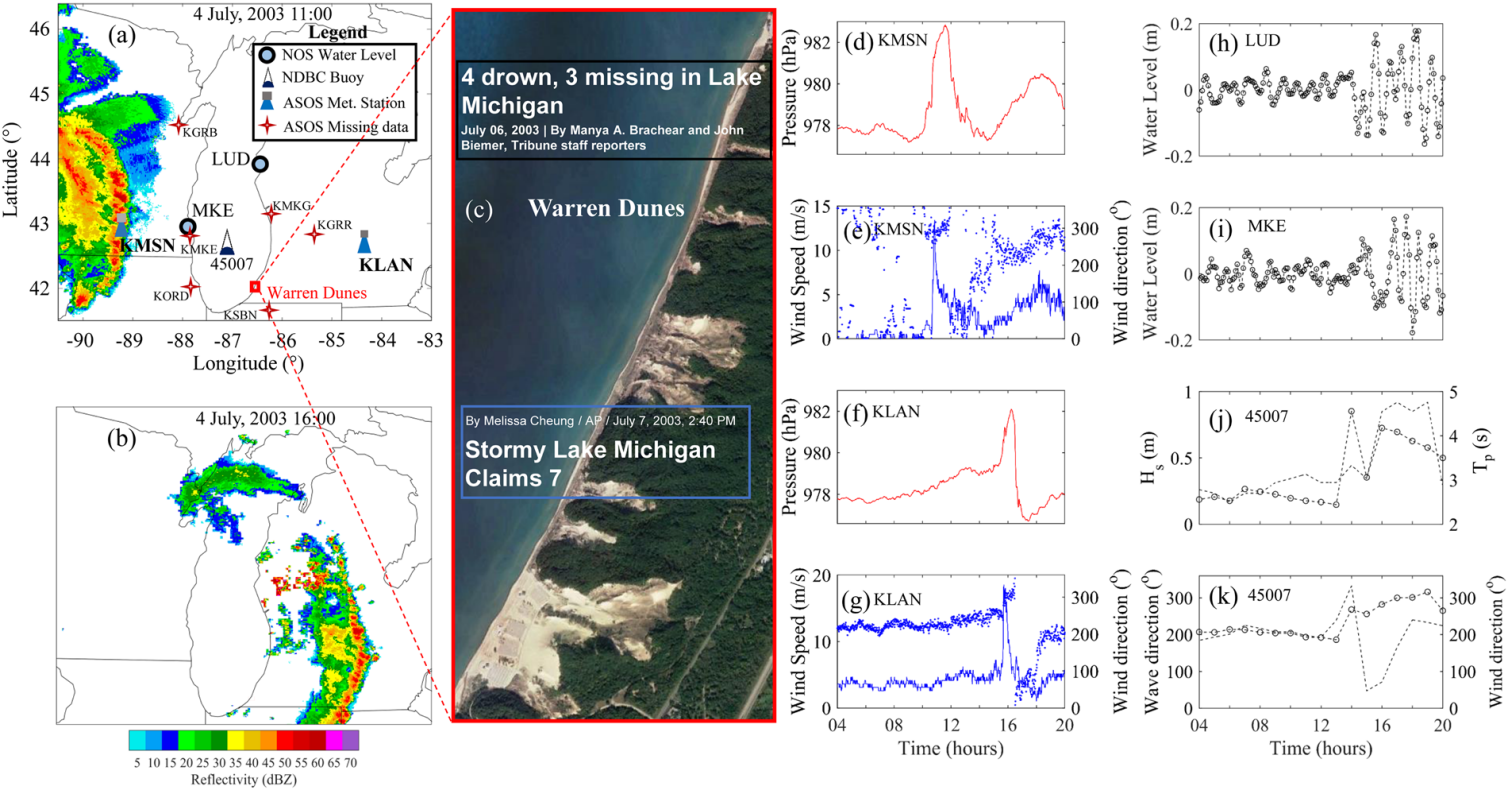
Observations on July 4, 2003. (**a**) and (**b**) are reflectivity imagery from the Iowa Environmental Mesonet NEXRAD Composite database at time 11:00 and 16:00, respectively. (**c**) A satellite image of WD (National Agriculture Imagery Program, image courtesy of the U.S. Geological Survey) and headlines of article news from (black) Chicago Tribune (www.chicagotribune.com) and (blue) CBS news (https://www.cbsnews.com); Observed air pressure (red), wind speed (solid blue), and wind direction (dot blue) at (**d**,**e**) KMSN and (**f**,**g**) KLAN; Water level at (**h**) LUD and (**i**) MKE; (**j**) Significant wave height (H_s_; dash-circle), peak wave period (T_p_; dashed line), and (**k**) peak wave direction (dash-circle) and wind direction (dashed line) at 45007 buoy. All times are in UTC. Wind and wave direction follow the nautical convention, i.e., the direction from where they propagate, measured clockwise from geographic North. Figure is created using MATLAB-2017a (http://www.mathworks.com/).

On July 4, 2003, seven people drowned near Warren Dunes (WD), a beach along the southeast shoreline of Lake Michigan (Fig. 1). Throughout most of the day at WD, the weather was sunny and warm, the wind was light, and the wave conditions were low. However, for less than 15 minutes, approximately between 1350–1405 UTC, a convective storm sweeping across Lake Michigan brought rain and wind gusts. Afterwards, the pleasant weather was restored and nearshore conditions became calm again. Surprisingly, the seven drownings occurred^1^ within a three-hour window in the afternoon (1630–1930 UTC). To date, the causes of the fatalities have been unknown though several conjectures have been made. First, a sudden water level rise caused by the storm could have washed beachgoers into the water by overtopping coastal structures in the area, similar to previous meteotsunami events in Lake Michigan^2,3^. Second, the swimmers could have been swamped by large wind waves, as some eyewitnesses reported wave heights above 1 m (*1*). Lastly, large wind waves could have generated dangerous rip currents that are commonly attributed to drowning accidents in Lake Michigan^4^. Despite all these conjectures, the cause of the seven drownings remains a mystery.

Meteotsunamis, different from seismically induced tsunamis, are meteorologically induced sub-basin propagating waves with periods ranging from a few minutes to 2 hours^5–7^. Meteotsunami formation is a multi-step process^5,8^. First, perturbations in wind stress and/or air pressure act on the water surface to initiate a meteotsunami wave. If the propagation speed of the atmospheric perturbations is close to the meteotsunami wave speed, the height of the meteotsunami wave resonantly grows^9^. Furthermore, the meteotsunami height can be enhanced through nearshore wave transformations such as shoaling, refraction, reflection, and superposition^10^. If the frequency of the wave is close to that of one of the modes of oscillations of a harbor or inlet, local resonance amplifies the meteotsunami height, potentially inundating shoreline areas or overtopping coastal structures to cause people injury and fatalities^11,12^. In the past, destructive meteotsunamis with wave heights of up to 3~6 m have been reported in areas of harbors or inlets^2,5,12^. Meteotsunamis with dangerous wave heights have also been observed along beaches. For instance, a meteotsunami of approximately 3 m height flooded Daytona Beach, FL, USA, on July 3, 1992, causing injuries to 75 individuals and damaging approximately 100 vehicles^13^. More recently, a meteotsunami ravaged some beach areas and injured 6 people in Odessa Beach, Ukraine, on June 27, 2014^14^. Overall, devastating meteotsunamis typically exhibit large wave heights that can cause damages, injuries, and fatalities in harbors, inlets, or beaches.

Rip currents are concentrated (10~100 m) and strong (>0.3 m/s) seaward flows^15^ that can extend beyond the surf zone. Rip currents have claimed hundreds of drownings and thousands of rescues worldwide every year^16,17^. One common generation mechanism of rip currents is the alongshore variations of breaking wave height^18,19^ due to the three-dimensional varying bathymetry^20–22^. There are two kinds of bathymetrically controlled rip currents^22^. Channel rips are caused by bathymetric anomalies consisting of deep channels between sandbars in the surf zone. Focused rips occur when the bathymetric anomalies are outside the surf zone with localized deeper areas that refract and focus waves in the surf zone. Another distinct mechanism is the alongshore variations of wave height and energy dissipation caused by the oblique incidence waves interacting with obstacles like natural headlands or man-made coastal structures. Deflecting/shadowing rips, known as boundary-controlled rip currents, are offshore flowing jets occurring on the front/lee side of the obstacle boundary^21^. Different from the previous mechanisms, hydrodynamically controlled rip currents^22^ are driven solely by hydrodynamic forcings in the absence of any morphological features. For example, shear instability rips are caused by instability of longshore currents in the cross-shore direction under oblique incident waves on longshore-uniform beaches^23,24^. To date, rip currents induced by meteotsunamis have not been reported.

The objective of this paper is to uncover the cause of the seven drownings on July 4, 2003 based upon a forensic science approach. Specifically, we compile all available data and eyewitness information to examine the causality of the event and employ state-of-art multi-scale hydrodynamic modeling to reconstruct the possible conditions near WD. Results, for the first time, uncover that the meteotsunami water level fluctuations and wind waves generated by a convective storm interacted with local bathymetry and coastal structures to initiate and modulate various types of rip currents. Meteorological tsunamis are revealed as a new generation mechanism of rip currents, thus posing an unexpected beach hazard that, to date, has been ignored.

## Results

Data forensics is used by compiling and synthesizing available atmospheric, wind wave, and water level observations to understand the conditions at the time of the event. Radar reflectivity imagery shows a bow-shaped convective storm^25^ crossing southern Lake Michigan with an eastward propagation direction (Fig. 1a,b). The storm propagated over the lake at approximately 29 m/s. Along the pathway of the storm, there were 8 weather stations, but 6 stations did not record any data during the event (Fig. 1a). Only two stations, KMSN and KLAN, recorded the fast-moving atmospheric perturbations at a temporal interval of 1 min. When the storm arrived at KMSN, the associated air pressure and wind abruptly increased 2.5 hPa and 13.7 m/s in 5 minutes (Fig. 1d,e). Once the storm reached KLAN, the pressure exhibited abrupt changes of 4.5 hPa magnitude over 10 minutes (Fig. 1f) and the wind intensified to 19 m/s (Fig. 1g). After the storm had passed, the direction of wind shifted from westerly to southeasterly (Fig. 1e,g,k).

Water level oscillations in Ludington (LUD) and Milwaukee (MKE) harbors had magnitudes of 0.34 m and 0.36 m, respectively, with periods close to 1 hour after the passage of the storm. The occurrence of water level oscillations with periods within the tsunami frequency band closely following a fast-moving storm with atmospheric perturbations crossing Lake Michigan indicate a meteotsunami event, similar to notable meteotsunamis in 1954 in Chicago, IL in Lake Michigan^3^ and 2012 in Cleveland, OH, in Lake Erie^10^. According to a recent study^7^, heights of water level oscillations of approximately 0.3 m are frequent in the Great Lakes. Given the low height of the meteotsunami, it is unlikely that the meteotsunami could have overtopped coastal structures to cause the 7 drownings near WD.

Wind wave conditions at a mid-lake buoy (Fig. 1j) were low, with significant wave heights (H_s_) and peak wave periods (T_p_) below 0.4 m and 3 s, respectively, before the arrival of the storm. Wave propagation direction (nautical convention) prior to the arrival of the storm was approximately 180° (northward), suggesting that H_s_ at WD was significantly smaller than the observed value at the buoy. The strong winds due to the storm increased H_s_ and T_p_ up to 0.85 m and 3.2 s at 1400 UTC, but H_s_ decreased to 0.4 m by 1500 UTC. Subsequently, wave propagation direction changed to 267° (Fig. 1k). In the later hours, H_s_ remained below 0.73 m with a nearly constant T_p_ close to 4.6 s. The magnitude of the relatively small wave heights is unlikely to directly cause multiple fatalities of swimmers^26^. Furthermore, wind waves of such heights and periods are rarely associated with rip-current drownings^17^. Note that all available wave climate and water level fluctuation data are tens of kilometers away from the location of this fatality event. As we seek to provide the best approximation as possible, the data forensics from these stations may not fully represent the actual conditions near WD.

**Figure 2:**
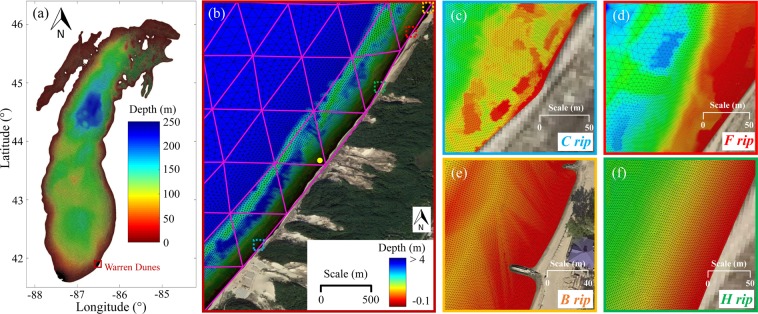
Bathymetry and unstructured meshes for hydrodynamic modeling. (**a**) Lake Michigan; (**b**) WD satellite image (National Agriculture Imagery Program, image courtesy of the U.S. Geological Survey). The purple mesh has a horizontal resolution of 600 m while the detailed mesh has horizontal resolution up to 2 m. Dashed boxes along the shoreline with different colors depict locations for 4 types of identified rip currents. The reference location with 1 m water depth for time series of Hs and Tp in Fig. 3 is depicted with a solid filled yellow circle. Zoom in at the dashed boxes from (**b**) where (**c**) channel (blue), (**d**) focused (red), (**e**) boundary-controlled (orange), and (**f**) hydrodynamically controlled (green) rip currents occur. Figure is created using MATLAB-2017a (http://www.mathworks.com/).

Integrated atmospheric-hydrodynamic modeling forensics using a coupled wave-current interaction model of Lake Michigan driven by a fast-moving storm event (Fig. 2) is employed to reconstruct the nearshore water conditions near WD. The integrated model has been validated (Fig. S1), showing excellent agreements between modeled and observed H_s,_ T_p_, and wave direction in buoy 45007 as well as water levels in the east and west coasts of Lake Michigan (Fig. S1). Modeling results indicate that the meteotsunami traveled eastward and was amplified in the center of the southern basin, where the wave speed (*c* = 28.8 m/s; depth~85 m) for non-trapped long waves was approximately equal to the storm propagation speed (U~29 m/s). The meteotsunami arrived at WD at 1405 UTC with periods and magnitudes of water level oscillations close to 95 min and up to 0.37 m, respectively (Fig. S2c–f). This moderate meteotsunami height at WD, similar to the values observed at LUD and MKE, is too low to overtop the coastal structures present to the north of WD (Fig. 2c). Heights of wind waves, H_s,_ at WD were approximately 0.45 m (Fig. S3a), calmer than the waves at the mid-lake buoy and also too low to cause any potential hazards. In addition, results obtained by the model with spatial grid resolutions of 600 and 30 m show the magnitudes of currents up to 0.03 and 0.1 m/s at WD, respectively (Fig. S2), which are unlikely to be responsible for the drownings. To this end, the modeling forensics based upon nearshore spatial grid resolutions of *O*(100 m) and *O*(10 m) does not present highly hazardous conditions that could have caused the seven drownings.

Detailed Nearshore Area (DNA) modeling forensics is employed by refining the model grid size with a magnitude of *O*(1 m) to disclose the appearance of rip currents, which have typical sizes of *O*(10 m) in the Great Lakes. Specifically, the nearshore grid size is enhanced up to 2 m to delineate the bathymetry and shape of coastal structures and shorelines provided by high-resolution lidar data (Fig. 2). Using intensive high-performance parallel computing, DNA modeling forensics reveals the presence of four types of rip currents near WD: (i) Channel (***C****) rips*; (ii) Focused (***F****) rips*; (iii) Boundary-controlled (***B****) rips*; and (iv) Hydrodynamically controlled (***H****) rips*.

**Figure 3:**
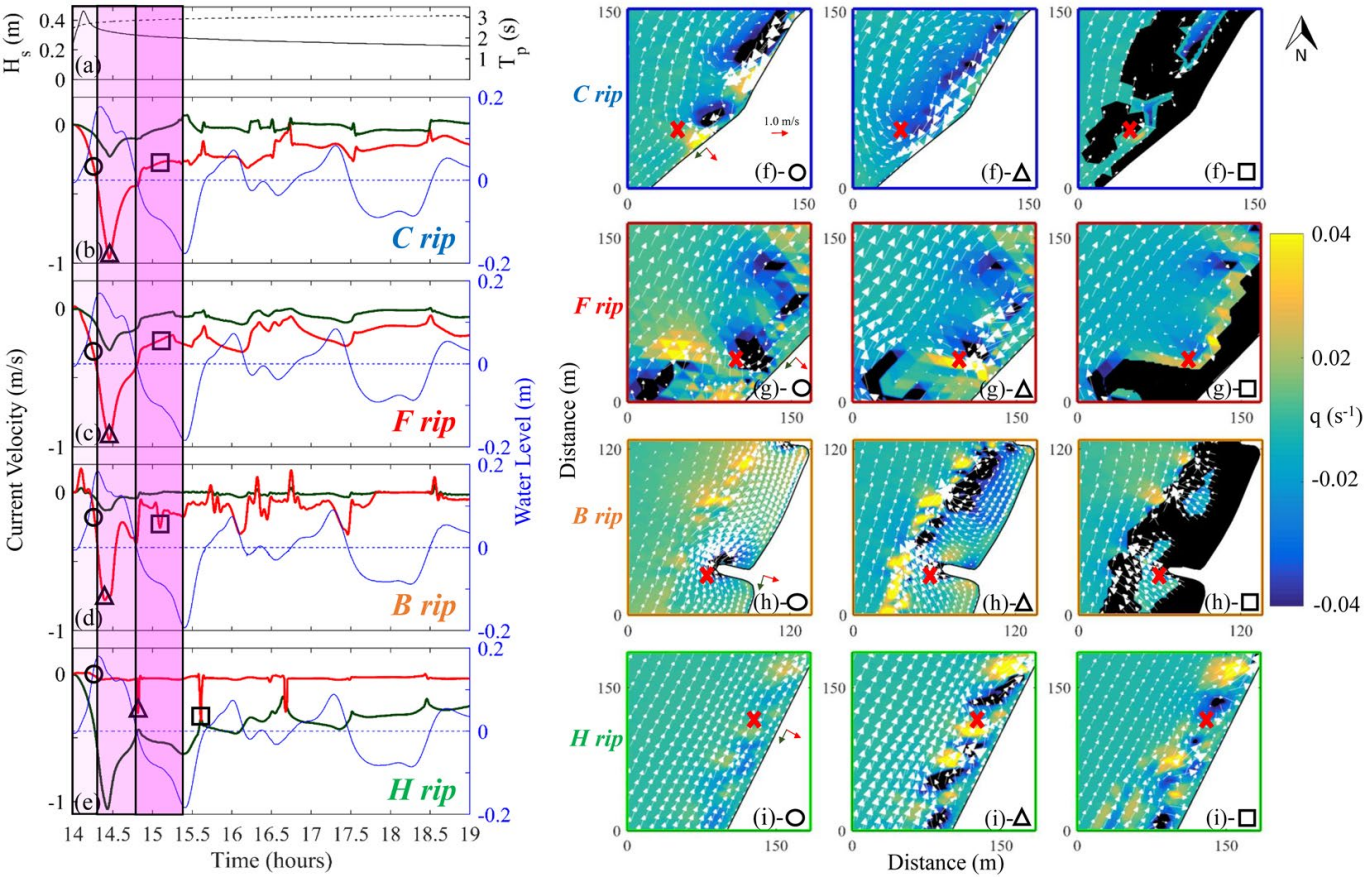
Reconstructed nearshore hydrodynamic conditions. (**a**) H_s_ (solid) and T_p_ (dashed) at 1 m water depth. (**b**–**e**) Time series of water level (blue) and cross-shore/longshore (red/green) velocities in the locations specified by a colored cross in the first column (circle) of vorticity and velocity plots. Positive directions for the time series of longshore/cross-shore velocities are specified with green/red arrows for each location in the circle column. Circle, triangle, and square shapes indicate the times when snap shots of vorticity and velocity vectors are taken at each of the 4 sites specified in Fig. 2. Black color in the third column (square) vorticity and velocity plots depicts dry areas.

Figure 3 shows the time series of water levels and current velocities for each rip type at representative locations near WD (Symbol x in red) and the time series of H_s_ and T_p_ at a reference location with 1 m water depth (Fig. 2b). Prior to the meteotsunami arrival, water conditions were relatively calm with H_s_ less than 0.1 m and current velocity below 0.05 m/s. The evolution of the storm-induced nearshore conditions can be divided into four distinct stages. During the meteotsunami runup stage between 1405 and 1420 h UTC (light magenta shaded area), H_s_ rapidly increased to the peak 0.45 m and then decreased to 0.35 m (solid line Fig. 3a) and T_p_ was 3 s (dashed line Fig. 3a). Water level rapidly increased from 0 m (mean lake level) to 0.18 m (blue lines Fig. 3b,e). Meanwhile the speed of cross-shore velocity increased from 0 to approximately 0.40 m/s, initiating ***C***, ***F***, and ***B**** rips* (red lines Fig. 3b,d with a negative value representing an offshore direction). In contrast to ***C***, ***F***, and ***B**** rips*, no ***H**** rip* appeared (red line Fig. 3e) but the speed of longshore velocity rose up to 0.6 m/s (green line Fig. 3e). Subsequently, during the early meteotsunami drawdown stage between 1420 and 1450 UTC (darker magenta shaded area), H_s_ gradually dropped to 0.29 m and T_p_ slightly increased to 3.05 s, suggesting an initiation of a swell. The rapidly receding water level due to the fast retreat of reflected meteotsunami waves at WD augmented the strengths of ***C***, ***F***, and ***B**** rips* with the speed ranging from 0.5 to 0.9 m/s for 30 minutes. By contrast, a ***H**** rip* occurred at the peak of the water level receding rate (1440 UTC) and persisted for only 2~3 minutes in the alongshore uniform beach while there was strong longshore velocity speeds of 0.45 m/s (Fig. 3e). During the later meteotsunami drawdown stage between 1450 and 1525 UTC (darkest magenta shaded area), the swell continuously evolved with H_s_ slowly dropping to 0.27 m and T_p_ slightly increasing to 3.1 s. The water level continuously receded to the lowest elevation during the event (−0.2 m below the mean), drying out the foreshore area and enlarging the beach area. ***C***, ***F***, and ***B**** rips* with a cross-shore current speed above 0.3 m/s maintained throughout this 35-minute period (Fig. 3b–d) but no ***H**** rip* occurred. After 1525 UTC (unshaded area), a seiching stage began when the meteotsunami wave reflected to the west side of Lake Michigan and rebounded back to WD. During this seiching stage, ***C***, ***F***, and ***B**** rips* were re-initiated and re-augmented as the reflected meteotsunami receded below the mean water level (1610 and 1730 UTC), with cross-shore current speeds exceeding a rip current threshold of 0.3 m/s. Similarly, ***H**** rips* reoccurred when water level from the reflected meteotsunami rose above the mean water level (1530 and 1640 UTC). Overall, DNA modeling forensics depicts many ***C***, ***F***, and ***B*** rips for hours and intermittent ***H**** rips* between 1 and 3 hours after the storm had passed.

Snapshots of the water velocity field superimposed on a vorticity color map for the four types of *rips* during the meteotsunami event at the representative locations are shown in Fig. 3. ***C**** rips*, identified by a pair of vortices are centered over surf zone sandbars, occurred at nine locations throughout the nearshore area near WD (Fig. S4). At the meteotsunami runup, two pairs of vortices were generated around the deeper channels located between sandbars (Fig. 2c) due to the difference in alongshore breaking wave heights, indicating the existence of two ***C**** rips* (Fig. 3f-○). At the early stage of the meteotsunami drawdown, the two pairs of vortices collapsed to form a meteotsunami-induced rip with a strong current up to −0.9 m/s (Fig. 3f-Δ). At the later stage of the meteotsunami drawdown, the water level retreated, drying out the foreshore area while the speed of the cross-shore return current decreased (Fig. 3f-□). ***F*** rips, identified by a pair of surf zone vortices generated by the focusing of wind waves refracted by bathymetric anomalies outside the surf zone, occurred at four locations near WD (Fig. S4). Figure 2d shows the two deeper regions (blue color) outside the surf zone at the top-left corner. While the direction of offshore wind waves at WD during the entire event varied between 270° and 340°, the nearshore waves were refracted to propagate perpendicular toward the coastline (Fig. S3). At the meteotsunami runup, refracted waves from the two deeper regions resulted in alongshore variable breaking heights, creating two opposing alongshore currents to meet and re-direct to offshore as an ***F*** rip (Fig. 3g-○). The strength of the rip current reached its maximum (−0.95 m/s) at the early stage of the meteotsunami drawdown (Fig. 3g-Δ) and decreased at the later stage of the drawdown (Fig. 3g-□). ***B**** rips*, identified by seaward currents, occurred at three locations where coastal structures or varying bathymetries interacted with the meteotsunami (Fig. S4). For example, Fig. 2e shows a 21 m-long coastal structure located to the north of WD. At the meteotsunami runup, wind waves and currents with a northeastward propagation interacted with the coastal structure, yielding a northwestward deflecting rip current on the windward side of the structure (Fig. 3h-○). At the early stage of the meteotsunami drawdown, the deflecting rip increased to the maximum speed (−0.8 m/s). In addition to the deflecting rip, a strong shadow rip at the leeward side of the structure appeared, leading to a clockwise vortex with large-scale outward velocities immediately behind the structure (Fig. 3h-Δ). At the later stage of meteotsunami drawdown, the retreat of water level dried out the foreshore and the flow field near the structure exhibited a cross-shore outward velocity (Fig. 3h-□). ***H**** rips* were identified in areas near WD with almost alongshore uniform bathymetry (Fig. S4) and lasted for a few minutes, in contrast to ***C***, ***F***, and ***B**** rips* that were relatively fixed in space and persistent in time. Figure 2f shows an alongshore-uniform stretch of beach with an average slope of 0.03. At the meteotsunami runup, the induced alongshore current interacted with the beach to generate the vorticity that tends to be dissipated by friction (Fig. 3i-○). As the water level rapidly decreased towards the minimum elevation, the vorticity cascaded to destabilize the alongshore current, yielding a ***H**** rip* lasting for 2~3 minutes (Fig. 3h-Δ). Approximately 40 to 60 minutes later, the water level rose due to the arrival of the reflected wave. When the change of water level reached to its peak, the longshore current underwent a rapid deceleration to generate another ***H**** rip* (Fig. 3i-□). Through the efforts of DNA modeling forensics, it is found that the horizontal grid resolutions is on the *O(1 m)*, *which can capture the size of* meteotsunami-induced rip currents with typical sizes of *O*(10 m).

How the meteotsunami generated the four types of rip currents is explained by revealing the dynamic evolution of water level fluctuations and nearshore current changes at WD, as shown in the supplemented four videos. For Video S1, the meteotsunami wave propagated eastward and reached the WD coastline obliquely, thus generating overall northward longshore currents. At the very nearshore at the north, refraction of the combination of meteotsunami waves and wind waves resulted in southward longshore currents, which were opposed by the northward longshore currents. As a result, the deflected cross-shore currents were funneled through the channels between sandbars to generate the ***C*** rip. The strength of the rip was greatly enhanced during the water level retreated period (1420–1430 UTC). Video S2 shows that the ***F*** rip, similar to the ***C*** rip, was the result of the deflected cross-shore focused currents due to meteotsunami wave refractions by the presence of deeper areas approximately 100 m away from the coastline. The strength of the ***F*** rip continued to increase during the arrival and reflection of the meteotsunami wave and reached to the maximum, (i.e.,~1 m/s) at 1425 UTC. Video S3 depicts the generation of two ***B*** rips at the windward or front side (shown as ●) and leeward or shadowed side (shown as ■) of the perpendicular coastal structure. During 1400–1414 UTC, the meteotsunami wave approached the WD and interacted with the structure to generate deflected and shadowed northward longshore currents. The deflected ***B*** rip at the windward side of the structure reached to the maximum during the water level retreated period (1420–1430 UTC). The shadow rip at the leeward side of the structure did not appear until the later water level retreated period (1430–1440 UTC). At last, Video S4 discloses an *H* rip on the alongshore-uniform beach with the absence of coastal structures. During the water level retreated period, the long-lasting longshore currents induced by the meteotsunami wave became unstable due to strong shear in the cross-shore direction, yielding unsteady propagating vortices. At 1443–1445 UTC, a short-live trainset ***H*** rip occurred due to vortices shed offshore. Overall, four different types of rip currents generated by a meteotsunami are clearly illustrated and explained by the supplemented videos in this paper for the first time, as far as the authors are aware.

## Discussion

The forensic science approach employed in this paper indicates that meteotsunami-induced rip currents are the most plausible cause of the 7 drownings on July 4, 2003. The above results, for the first time, uncover that the meteotsunami water level fluctuations and wind waves generated by a convective storm interacted with local bathymetry and coastal structures to initiate the various types of rip currents. The generated rip currents can change the nearshore current conditions from calm to hazardous in a few minutes due to the nature of fast-moving convective storms induced rapid changes in water level. This hidden hazard can last for several hours, even under subsequent low energy wind wave conditions. Similar to tides^20^, meteotsunamis can modulate the temporal and spatial characteristics of different types of rip currents, dangerously broadening the range of nearshore areas that can lead to unexpected hazardous rip currents.

**Figure 4:**
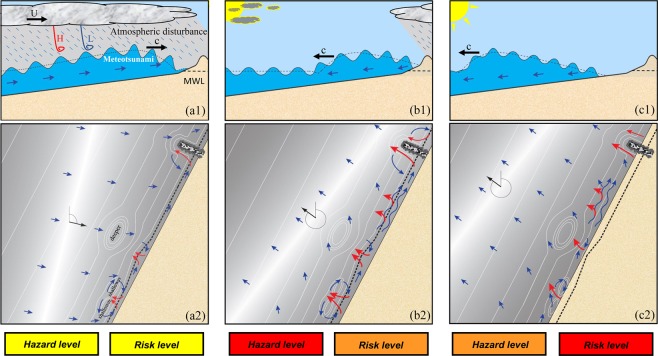
Hazard and risk levels of the three stages during which the meteotsunami generated and modulated rip currents. Schematic (**1**) is side view of the meteotsunami wave, wind waves, atmospheric conditions, and schematic (**2**) plan view of currents, and water level during the (**a**) meteotsunami runup, (**b**) early meteotsunami drawdown, and (**c**) later meteotsunami drawdown. Rip currents are depicted in red arrows and water levels are represented by a grayscale map with white being largest. The shoreline at mean water level (MWL) is shown as a black dashed line. Hazard and risk levels associated with each stage with yellow color being the lowest, orange being the intermediate, and red being the highest, are shown at the bottom.

The hazard and risk levels for the three stages of meteotsunami impacts are categorized based upon the average speed of rip currents and the likelihood that they will impact swimmers (Fig. 4). The hazard level, represented by rip current speed, was the lowest at the meteotsunami runup while during the early meteotsunami drawdown was the highest. The risk level, or likelihood of drowning due the presence of rip currents combined with the perception and presence of beachgoers that expose them to rip current dangers, can be different and even change depending on education and communication of meteotsunami hazards^15^. On July 4, 2003, the fast convective storm reached WD simultaneously with the meteotsunami runup (Fig. 4a). The wind waves were relatively small, thus vulnerability to the hazard was low. The rainfall and strong wind were unappealing for beachgoers, thus reducing the number of swimmers and leading to a low risk classification. Only 15 minutes later, the storm and the associated wind gusts had ceased. Meteotsunami drawdown initiated and rip currents were generated (Fig. 4b), increasing the vulnerability to yield the highest hazard. Meanwhile, most beachgoers had not re-enter the water around that time so the risk level was intermediate. At the end of meteotsunami drawdown and well after the passage of the storm, beachgoers who had been waiting for the storm to pass as well as new beachgoers that may have arrived due to the nice weather were likely to enter the nearshore water. The combination of the increase in beach users and the moderate yet unexpected hazard of meteotsunami-induced rips elevated the risk to the highest level (Fig. 4c). The temporal difference between hazard and risk levels in this event likely explains why all the drownings occurred after the passage of the storm. This paper for the first time elucidates different degrees of beach hazard and risk levels associated with the three stages of water and weather conditions due to a fast-moving storm as well as unexpected rips generated by a meteotsunami.

How often may dangerous rips have been generated by meteotsunamis in the Great Lakes? Given the previously unknown knowledge and connection between meteotsunamis and nearshore rips, historical records from the Great Lakes Current Incident Database (GLCID)^4^ are compiled and deduced to address the above question. Eyewitness incident reports show that rip currents have led to 94 fatalities and 298 rescues at Lake Michigan beaches over the last 15 years. We cross-compare the occurrence dates of current-related incidents^4^ with those of meteotsunami events in Lake Michigan^25^. 16% of fatal current incidents and 12% of reported rescues occurred on the same day that meteotsunamis were observed in the lake. Table 1 lists these events with the associated H_s_ and meteotsunami heights H_m_. The coincident occurrence of meteotsunamis and water-safety incidents suggests that meteotsunami-induced rips are not sporadic but may be frequently related to each other. Unlike most destructive meteotsunamis reported in the literature, the heights of meteotsunamis associated with fatal rip-current incidents were below 0.5 m with an average of 0.3 m. Recent meteotsunami climatological studies^7^ have shown that meteotsunamis with heights above 0.3 m are frequently observed in the Great Lakes and in most regions of the world ocean^6^. Given the frequent occurrence of moderate-height meteotsunamis, the co-occurrence of rip-current fatalities suggests that meteotsunamis may pose a previously unrecognized safety hazard to coastal communities. Therefore, a crucial need is called for forecasts, education, and communication of meteotsunamis as well as meteotsunami-induced rip currents.

**Table 1:** Fatal or rescued current-related events in Lake Michigan (GLCID) that occurred the same day of a meteotsunami.

Date	Fatalities	Rescues	Beach Name, State	H_s_ (m)	H_m_ (m)
6/11/2002	0	1	Grand Haven State Park, MI	0.9–1.2	0.32
7/4/2003	7	0	WD State Park & Adjacent Beaches, MI	0.9–1.2	0.36
7/18/2006	1	0	Lions Park Beach, MI	0.9–1.2	0.43
6/13/2008	0	1	Silver Beach, MI	0.9–1.2	0.45
7/17/2008	1	0	Pierce Street Beach, MI	0–0.6	0.153
6/6/2010	0	2	Beverly Shores, IN	0.9–1.2	0.51
8/19/2010	1	0	Grand Haven State Park, MI	0.9–1.2	0.23
5/22/2011	1	0	Silver Beach, MI	0.9–1.2	0.265
6/8/2011	0	3	Manistique Beach, MI	0.9–1.2	0.27
6/19/2012	1	0	Silver Beach, MI	0.9–1.2	0.42
7/7/2012	0	21	Holland State Park, MI & South Haven Beach, MI	1.5–1.8	0.15
6/27/2016	0	2	Washington Park Beach, IN	0.9–1.2	0.17
8/31/2016	2	2	Holland State Park, MI	1.5–1.8	0.29
5/16/2017	1	0	Rogers Park Beach, IL	0–0.6	0.25
7/7/2017	0	4	Stearns Park, MI	0.9–1.2	0.19

H_s_ are provided by the GLCID. Maximum meteotsunami height (H_m_) is obtained from the NOAA/NOS station closer to the current-related event.

## Methods

Radar reflectivity composite imagery from the Iowa Environmental Mesonet is used to depict the spatial structure of the storm with 1 km spatial resolution at 5 min intervals. Air pressure and wind speed at 1 min resolution are obtained from National Weather Service (NWS) Automated Surface Observing System (ASOS) at two locations; Madison (KMSN) and Lansing (KLAN) airports. Other ASOS stations failed gathering data during the event, making KMSN and KLAN the only two stations with air pressure and wind speed data during the passage of the storm. Water level observations obtained at 6 min sampling frequency are obtained at Milwaukee (MKE) and Ludington (LUD) from stations operated by the National Oceanic and Atmospheric Administration (NOAA) National Ocean Service (NOS). Significant wave height (H_s_), peak wave period (T_p_) and wave direction in the southern Lake Michigan basin are obtained from the offshore buoy 45007 operated by the NOAA National Data Buoy Center (NDBC).

The atmospheric forcing used to the Integrated atmospheric-hydrodynamic modeling consists of wind and air pressure perturbations traveling from west to east at 29 m/s superimposed to the interpolated wind conditions from NOAA Great Lakes Coastal Forecasting System (GLCFS)^27^. Atmospheric perturbations are created with a temporal resolution of 1 min. Spatially, perturbations are assumed to have uniform bandwidth and constant speed and direction, which is a common assumption in meteotsunami modeling studies^3,28,29^. Similar to observations in KMSN and KLAN, the perturbations used as forcing exhibit a trapezoidal shape and the following characteristics: Wind speed rises from 0 to 14 m/s in 5 minutes, maintains 14 m/s during 3 minutes, and drops back again to 0 m/s in 15 minutes. Pressure increases from 0 to 2.5 hPa in 5 minutes, it is maintained constant at 2.5 hPa for 60 minutes, and it drops back again to 0 hPa in 10 minutes. While GLCFS wind conditions have a temporal resolution of 1 hour, GLCFS wind conditions are essential to recreate the existent wind wave conditions before and after the passage of the storm.

The 3^rd^ generation spectral Wind Wave Model (WWM-III)^30^ coupled with the Semi-implicit Cross-Scale Hydroscience Integrated System Model (SCHISM)^31,32^, that solves the 3D shallow-water equations with hydrostatic and Boussinesq approximations, is used to reconstruct the nearshore hydrodynamics in WD during the event. The wave-current interaction model is based on unstructured grids, being suitable for high-resolution multiscale studies^30,33^. Specifically, the coupled model (WWMIII-SCHISM) has been employed in the past to study storm surges^33^ and inundation^34^. SCHISM has been used to study tsunamis^35,36^ and meteotsunamis^3,8,37^. In this study, we use WWMIII-SCHISM to reconstruct the wind wave and current conditions during the event. A high-resolution unstructured grid (548,000 elements), with grid size ranging from 2 m in WD to 600 m in the center of the lake (Fig. 2), is constructed to represent Lake Michigan and WD in detail (Fig. 2b). Specifically, the depth of the WD nearshore grid is interpolated using high-resolution lidar bathymetry (970,000 points) downloaded from https://maps.ngdc.noaa.gov/viewers/bathymetry/. The vertical domain is divided in 20 layers of terrain-following coordinates. A time step of 3 s is used to meet the stability conditions of the coupled model. The exchange of information between models is conducted every time step to capture the rapidly varying current and wave conditions in WD. This exchange consists of passing the radiation stress, total surface stress, and wave orbital velocity to SCHISM and passing water level, velocity, and wet/dry flags to WWMIII. The exchange of information every time step when using the radiation stress approach is essential to accurately model the wind wave-induced vorticity, and hence, rip currents^38^. Furthermore, temporal variations of the shoreline are treated with a shoreline tracking algorithm to capture water level oscillations induced by wind waves and a tsunami-like wave. This technique, advocated by the tsunami model known as MOST, requires high spatial and temporal resolutions, providing high accuracy in complex geometries^39^. Overall, a high-resolution wave-current interaction model is used to reconstruct the wind wave and current conditions during the July 4, 2003 event. To the author’s knowledge, this is the first time that a wave-current interaction model is employed to investigate the combined role of wind waves and a tsunami-like wave on nearshore currents.

Comparison between observed and modeled water level at LUD and MKE, and wind wave conditions at the 45007 buoy location is shown in Fig. S1. Specifically, in LUD, maximum wave height of 0.34 m and a period of approximately 1 hour is captured by the model during the first 3 hours after the meteotsunami arrival (Fig. S1a). In the following hours, modeling results exhibit a significant reduction in the amplitude of the oscillations in comparison with observations. This discrepancy is caused by the reason that the model grid domain does not extend to the elongated LUD harbor where the water level gage is located. Instead, the model gage is located in Lake Michigan coastline at the latitude of LUD. As a result, the model captures the beginning of water level fluctuations but does not reproduce later oscillations at the station LUD. In contrast, MKE exhibits a wide-open shape which is less prone to modulate long-period waves, leading to an excellent agreement between observed and modeled water level amplitude and period during several hours (Fig. S1b). The model also captures reasonably well H_s_, T_p_, and wave direction (Fig. S1c,d). Specifically, the rapid increase in H_s_ and wave directions during the passage of the storm is well modeled. Furthermore, results also show good agreement in T_p_ during the simulation. Overall, the excellent agreement between observed and modeled water level and wind wave conditions validates the integrated atmospheric-hydrodynamic modeling with the reconstructed atmospheric forcing for the event.

## Supplementary information

Supplemental Information

Formation of channel rips

Formation of focused rips

Formation of boundary-controlled rips

Formation of hydrodynamically controlled rips

## Data Availability

Meteorological data from ASOS stations (https://www.faa.gov/air_traffic/weather/asos/). Water level data from NOAA Tides & Currents (https://tidesandcurrents.noaa.gov/). Wave climate data from NOAA NDBC (https://www.ndbc.noaa.gov/station_page.php?station = 45007). Radar reflectivity imagery from IOWA Mesonet (https://mesonet.agron.iastate.edu/archive/data/). Lidar bathymetry from NOAA- NCEI (https://maps.ngdc.noaa.gov/viewers/bathymetry/). Image of WD from USGS Earth Explorer (https://earthexplorer.usgs.gov/).
