# Supplementary material for: Unexpected rip currents induced by a meteotsunami
Source: Sci Rep. 2019 Feb 14;9:2105. doi: 10.1038/s41598-019-38716-2 (PMC6376005; doi:10.1038/s41598-019-38716-2)
Supplement: Supplementary file 1 — Supplemental Information [file 41598_2019_38716_MOESM1_ESM.doc]

Supplemental Information for

**Unexpected rip currents induced by a meteotsunami**

Álvaro Linares, Chin H. Wu^*^, Adam J. Bechle, Eric J. Anderson, David A. R. Kristovich

^*^ Correspondence to: chinwu@engr.wisc.edu

^1^Department of Civil and Environmental Engineering, University of Wisconsin-Madison, Madison, WI, USA

^2^Wisconsin Sea Grant Institute, University of Wisconsin-Madison, Madison, WI, USA

^3^National Oceanic and Atmospheric Administration, Great Lakes Environmental Research

Laboratory, Ann Arbor, MI, USA

^4^ISWS, Prairie Research Institute, University of Illinois at Urbana-Champaign, Urbana, IL, USA

**Summary**

Supporting figures consists of FigS1 is the comparison of model results and observations for water levels, wave height, wave period, and wave direction; Fig. S2 is morphology, bathymetry and unstructured mesh of WD with approximately 600 m resolution and time series of model results for wave height, wave periods, and water levels; Fig. S3 is snapshots of significant wave height (H_s_) and peak wave direction (depicted with black arrows) at WD; and Fig S4 shows the locations where rip currents occurred near WD between 1405 and 1900 UTC on July 4, 2003.

**Figure Legends**

**Fig. S1.** Observed (circle and dashed line) and modeled (solid line) water level at **(a)** LUD and **(b)** MKE, **(c)** H_s_ (black) and T_p_(blue), and **(d)** peak wave direction at buoy 45007.

**Fig. S2. (a)** Morphology, bathymetry and unstructured mesh of WD. The purple mesh has a horizontal resolution of approximately 600 m while the detailed mesh has horizontal resolution up to 30 m. Circles indicate the locations used to obtain the time series of the 30 m grid. Time series of **(b)** H_s_ (solid) and T_p_ (dashed) at 1 m depth and **(c - e)** water level (blue) and cross-shore/ longshore (red/green) velocities for the 30 m (solid line) and 600 m (dotted line) grid. Time series corresponding to the 600 m grid are obtained from the nodes closest to the colored circles in **A**. The satellite image of WD is obtained from the National Agriculture Imagery Program, image courtesy of the U.S. Geological Survey. Figure is created using MATLAB-2017a (http://www.mathworks.com/).

**Fig. S3.** Snapshots of significant wave height (H_s_) and peak wave direction (depicted with black arrows) at WD during the event for WD (same area shown in Fig 2b). All times are in UTC.

**Fig. S4.** Locations where rip currents occurred near WD between 1405 and 1900 UTC on July 4, 2003. The satellite image of WD is obtained from the National Agriculture Imagery Program, image courtesy of the U.S. Geological Survey. Figure is created using MATLAB-2017a (http://www.mathworks.com/).


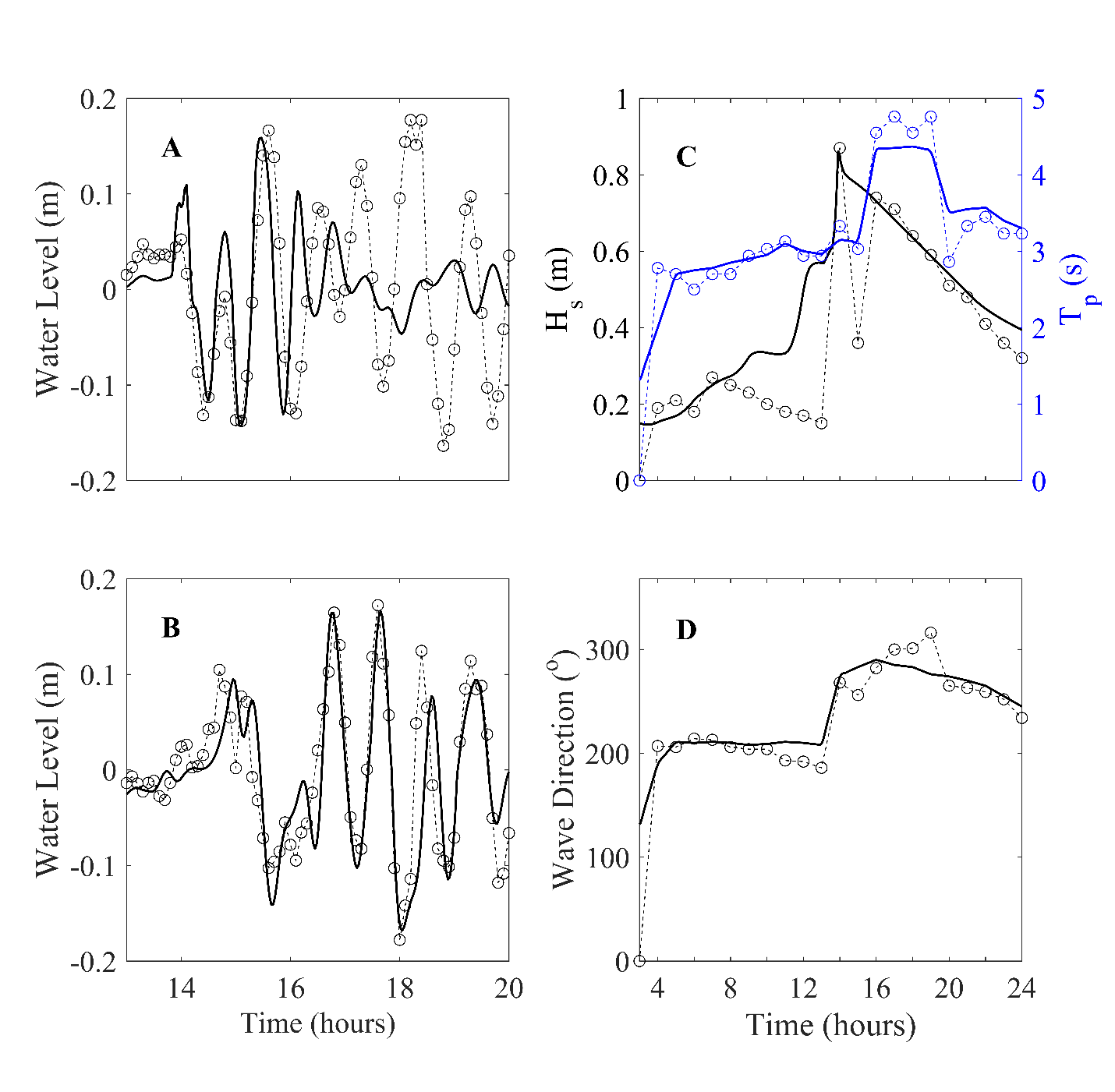


(a)

(b)

(c)

(d)

Fig. S1. Observed (circle and dashed line) and modeled (solid line) water level at (a) LUD and (b) MKE, (c) H_s_ (black) and T_p_(blue), and (d) peak wave direction at buoy 45007.

**
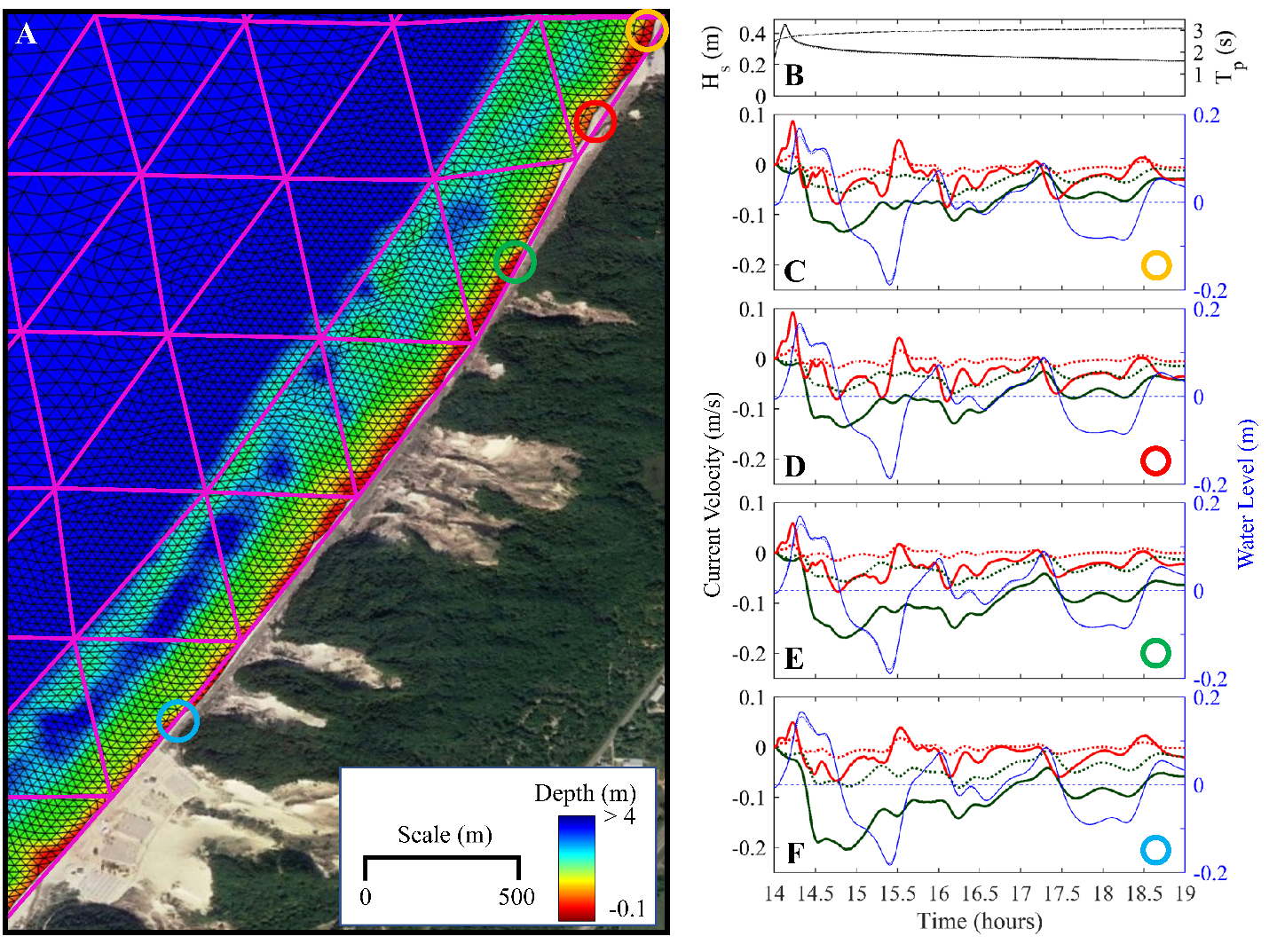
 Figure S2.** (**a**) Morphology, bathymetry and unstructured mesh of WD. The purple mesh has a horizontal resolution of approximately 600 m while the detailed mesh has horizontal resolution up to 30 m. Circles indicate the locations used to obtain the time series of the 30 m grid. Time series of (**b**) H_s_ (solid) and T_p_ (dashed) at 1 m depth and (**c** - **f**) water level (blue) and cross-shore/ longshore (red/green) velocities for the 30 m (solid line) and 600 m (dotted line) grid. Time series corresponding to the 600 m grid are obtained from the nodes closest to the colored circles in (**a**). The satellite image of WD is obtained from the National Agriculture Imagery Program, image courtesy of the U.S. Geological Survey. Figure is created using MATLAB-2017a (http://www.mathworks.com/).

(a)

(b)

(c)

(d)

(e)

(f)


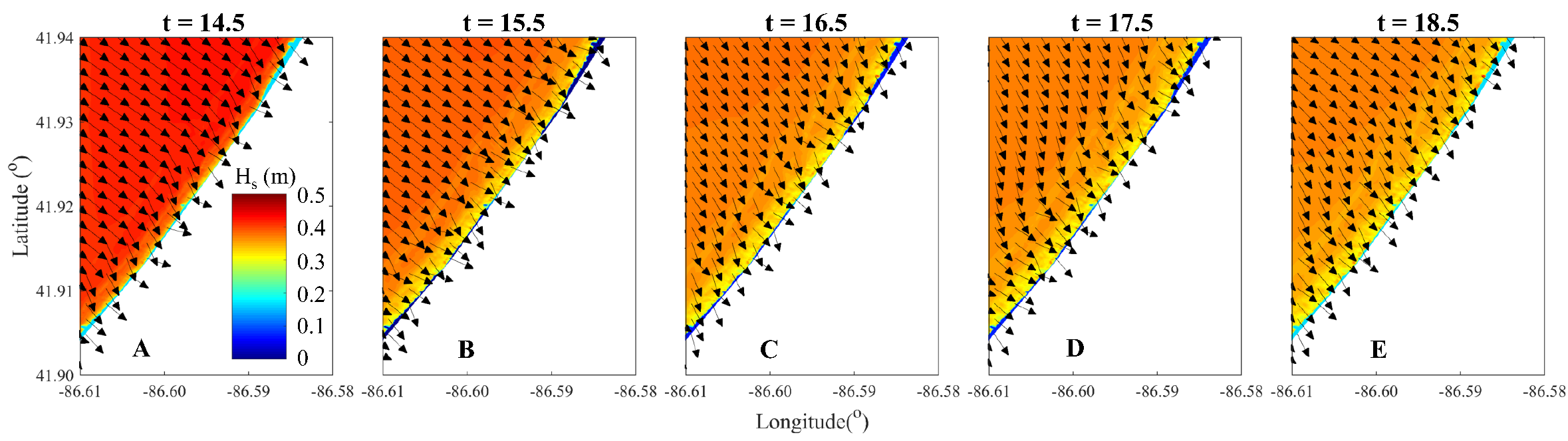


(a)

(b)

(c)

(d)

(e)

**Fig. S3**. Snapshots of significant wave height (H_s_) and peak wave direction (depicted with black arrows) at WD during the event for WD (same area shown in Fig 2B). All times are in UTC.

**
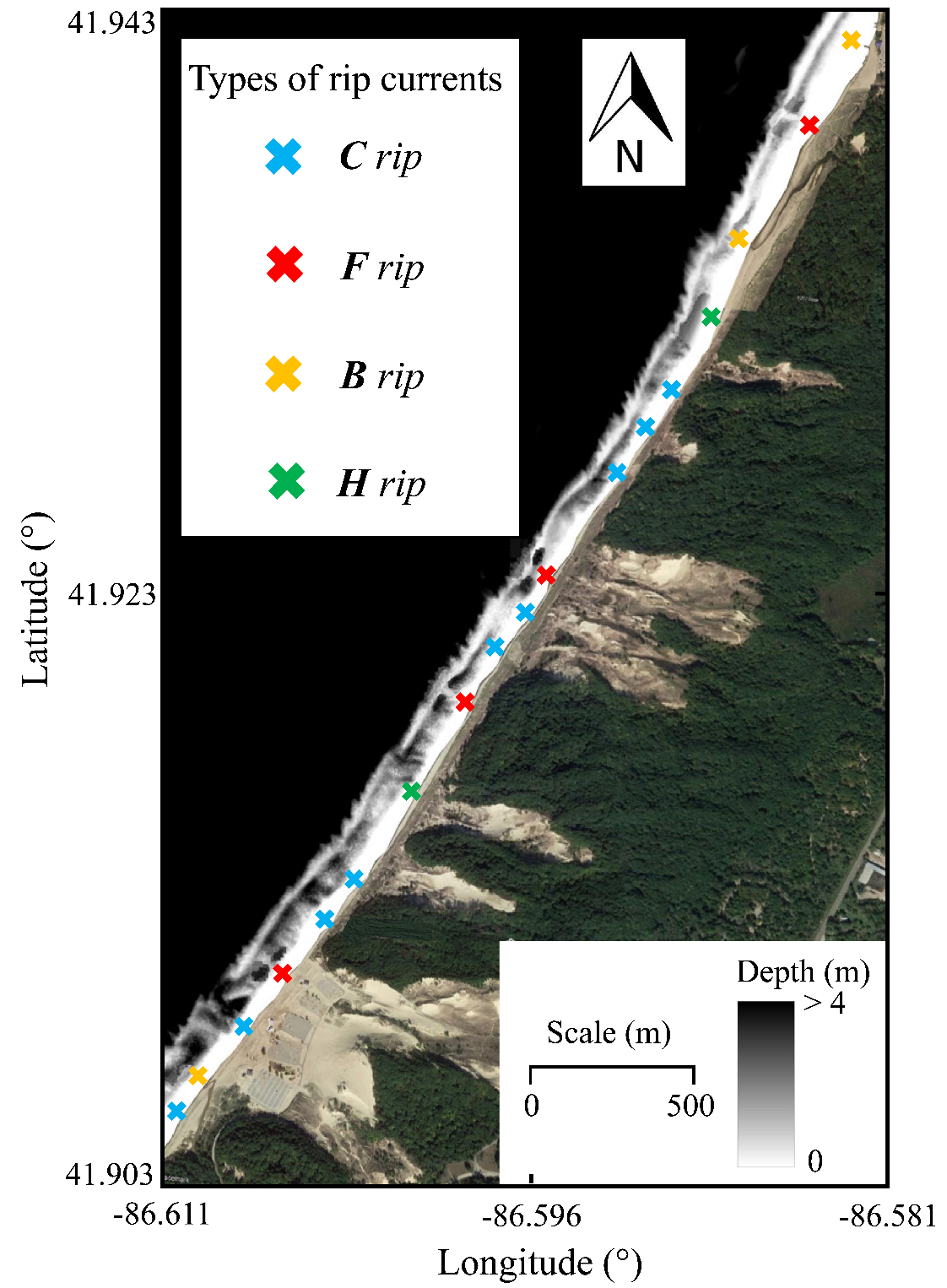
**

**Fig. S4**. Locations where rip currents occurred near WD between 1405 and 1900 UTC on July 4, 2003. The satellite image of WD is obtained from the National Agriculture Imagery Program, image courtesy of the U.S. Geological Survey. Figure is created using MATLAB-2017a (http://www.mathworks.com/).

**Supplementary video legends**

Video S1.mp4

Title: Formation of channel rips

Legend: Animation of the formation of channel rips by the meteotsunami between 1400 and 1445 UTC based on the numerical reconstruction of the event. Spatial map of water level and velocities is depicted on the right-hand side. Time series of water level (top) and speed of rip currents (bottom) at the location specified by a white circle are depicted on the left-hand side. The spatial domain is identical as depicted in Fig. 2 and Fig. 3. The video is created using MATLAB-2017a (http://www.mathworks.com/).

Video S2.mp4

Title: Formation of focused rips

Legend: Animation of the formation of focused rips by the meteotsunami between 1400 and 1445 UTC based on the numerical reconstruction of the event. Spatial map of water level and velocities is depicted on the right-hand side. Time series of water level (top) and speed of rip currents (bottom) at the location specified by a white circle are depicted on the left-hand side. The spatial domain is identical as depicted in Fig. 2 and Fig. 3. The video is created using MATLAB-2017a (http://www.mathworks.com/).

Video S3.mp4

Title: Formation of boundary-controlled rips

Legend: Animation of the formation of boundary-controlled rips by the meteotsunami between 1400 and 1445 UTC based on the numerical reconstruction of the event. Spatial map of water level and velocities is depicted on the right-hand side. Time series of water level (top) and speed of rip currents (bottom) at the location specified by a white circle and white square are depicted on the left-hand side as circles and squares, respectively. The spatial domain is identical as depicted in Fig. 2 and Fig. 3. The video is created using MATLAB-2017a (http://www.mathworks.com/).

Video S4.mp4

Title: Formation of hydrodynamically controlled rips

Legend: Animation of the formation of hydrodynamically controlled rips by the meteotsunami between 1435 and 1450 UTC based on the numerical reconstruction of the event. Spatial map of water level and velocities is depicted on the right-hand side. Time series of water level (top) and speed of rip currents (bottom) at the location specified by a white circle are depicted on the left-hand side. The spatial domain is identical as depicted in Fig. 2 and Fig. 3. The video is created using MATLAB-2017a (http://www.mathworks.com/).
